# A Case of Peritoneal Dialysis-Related Peritonitis Caused by Kocuria rhizophila

**DOI:** 10.7759/cureus.74856

**Published:** 2024-11-30

**Authors:** Kenta Torigoe, Ai Yoshidome, Kanoko Ashizawa, Haruka Fukuda, Emiko Otsuka, Kiyokazu Tsuji, Ayuko Yamashita, Mineaki Kitamura, Takahiro Takazono, Noriho Sakamoto, Kumiko Muta, Hiroshi Mukae, Tomoya Nishino

**Affiliations:** 1 Department of Nephrology, Nagasaki University Hospital, Nagasaki, JPN; 2 Respiratory Medicine, Nagasaki University Graduate School of Biomedical Sciences, Nagasaki, JPN

**Keywords:** end stage renal disease (esrd), kocuria rhizophila, peritoneal dialysis complication, peritoneal dialysis (pd), peritoneal dialysis-related peritonitis

## Abstract

A 63-year-old woman undergoing peritoneal dialysis (PD) presented to our hospital with abdominal pain, diarrhea, and cloudy PD effluent. An elevated white blood cell count in the PD effluent led to a diagnosis of PD-associated peritonitis. She was subsequently started on intraperitoneal cefazolin and ceftazidime, after which her condition improved rapidly. The peritonitis resolved after 21 days of therapy, with no subsequent relapse. Centrifuged PD effluent samples identified *Kocuria rhizophila* as the causative organism. Reports of PD-associated peritonitis caused by *K. rhizophila* are rare, with reported adult cases requiring PD catheter removal due to relapse. In contrast, this case was successfully resolved without catheter removal. The increasing use of advanced technologies, such as matrix-assisted laser desorption/ionization time-of-flight mass spectrometry, is expected to lead to more reports of *K. rhizophila*-associated PD peritonitis in the future. Although biofilm formation by *K. rhizophila* is known to increase the risk of recurrent peritonitis, this case suggests that catheter removal may not always be necessary.

## Introduction

Peritoneal dialysis (PD)-associated peritonitis is a significant complication in patients undergoing PD, which can lead to the discontinuation of the treatment. Effective treatment requires the selection of antibiotics based on the causative organism, and in cases of refractory or relapsing infections, removal of the PD catheter is often necessary [1]. While various factors contribute to refractory or relapsing PD-associated peritonitis, specific bacterial species are known to carry a higher risk [2].

*Kocuria rhizophila* is a Gram-positive coccus belonging to the family Micrococcaceae within the Actinobacteria phylum. It has been isolated from diverse natural sources, including freshwater, soil, fermented foods, sludge, and fish intestines [3]. Reports of PD-associated peritonitis caused by *K. rhizophila* are rare, with most cases requiring PD catheter removal for resolution [4,5]. Here, we present a case of *K. rhizophila*-associated PD peritonitis successfully treated without PD catheter removal.

## Case presentation

We report the case of a 63-year-old Japanese woman who began PD one year prior due to end-stage renal disease (ESRD) caused by chronic glomerulonephritis. She had no history of PD-associated peritonitis. She had experienced diarrhea for two months before admission, and on the day before admission, she noticed cloudy PD effluent. The following day, she developed abdominal pain with an increased frequency of diarrhea; therefore, she visited our hospital.

**Figure 1:**
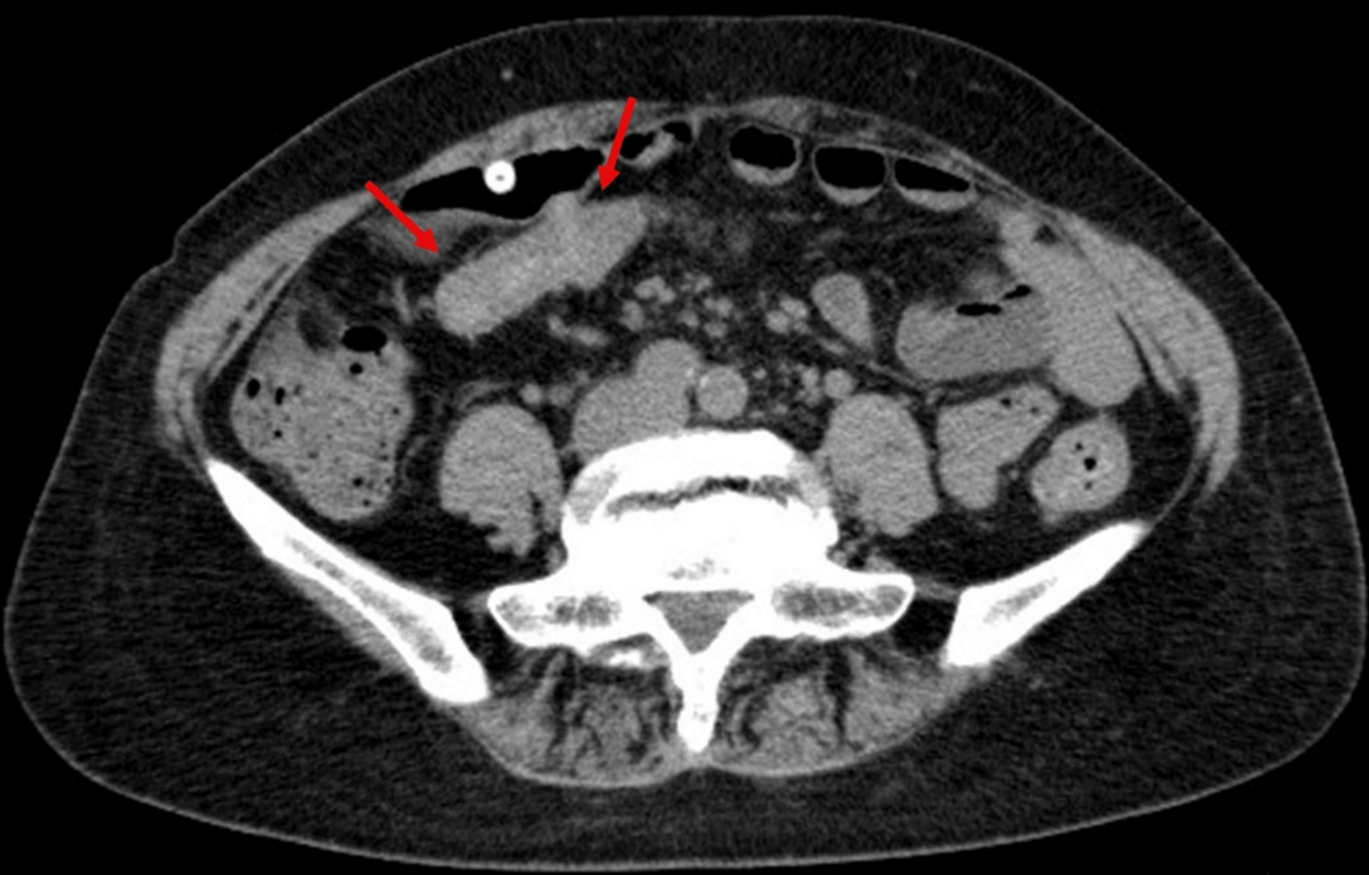
Abdominal CT findings. Edematous thickening is observed in the transverse colon (arrows). CT: computed tomography.

Analysis of the PD effluent revealed an elevated white blood cell count of 500/μL (differential leukocyte counts were unavailable due to emergency processing), leading to a diagnosis of PD-associated peritonitis. The patient was subsequently hospitalized. Her vital signs were stable at admission: body temperature 36.7°C, blood pressure 118/73 mmHg, pulse rate 61 bpm, and SpO₂ 96% (on room air). Her abdominal pain disappeared. Physical examination revealed no abdominal tenderness or signs of infection, such as redness or pain, at the PD catheter exit site or tunnel. Laboratory tests revealed a normal peripheral white blood cell count (5800/μL, normal range 3300-8600/μL) but mildly elevated C-reactive protein (CRP) levels (1.01 mg/dL, normal range 0.00-0.14 mg/dL). Abdominal computed tomography (CT) imaging revealed edematous thickening of the transverse colon, suggesting colitis (Figure 1).

The patient was started on intraperitoneal cefazolin (1 g/day) and ceftazidime (1 g/day) according to the International Society for Peritoneal Dialysis (ISPD) guidelines [1]. Following the initiation of treatment, her diarrhea improved rapidly. By the third day of hospitalization, the cloudiness in the PD effluent had cleared, and the white blood cell count in the PD effluent had decreased to 100/μL. Given her stable condition, she was discharged on day 7 and continued intraperitoneal antibiotic therapy as an outpatient.

PD effluent cultures collected at admission (without centrifugation) were negative; therefore, the causative organism of the PD-associated peritonitis remained unknown at discharge. However, subsequent analysis of a centrifuged PD effluent sample collected at admission, following the ISPD guidelines [1], identified *K. rhizophila* using matrix-assisted laser desorption/ionization time-of-flight (MALDI-TOF) mass spectrometry (Table 1). Since bacterial identification took time and the patient had responded well to the initial therapy with cefazolin and ceftazidime, no changes to the antibiotic regimen were made. A total of 21 days of antibiotic therapy was completed. The patient has been followed up without any recurrence.

**Table 1:** Drug susceptibility test results for Kocuria rhizophila. MIC: minimum inhibitory concentration.

Antimicrobial agent	MIC (mg/mL)
Oxacillin	>4
Ampicillin	≤0.5
Tazobactam/piperacillin	≤0.5
Cefazolin	2
Cefoxitin	≤0.5
Imipenem	≤0.5
Meropenem	≤0.5
Arbekacin	≤0.12
Erythromycin	≤0.25
Clindamycin	≤0.25
Minocycline	≤0.5
Vancomycin	≤0.5
Teicoplanin	≤0.5
Levofloxacin	1
Trimethoprim-sulfamethoxazole	≤1
Linezolid	≤1
Daptomycin	≤0.5

## Discussion

We present a case where *K. rhizophila*-associated PD peritonitis was cured without PD catheter removal, which is in contrast to previously reported cases. The cornerstone of PD-associated peritonitis management is antibiotic therapy; however, refractory or relapsing cases often necessitate PD catheter removal [1]. Various factors contribute to refractory or relapsing peritonitis, with the causative bacterial species being a critical determinant [1]. Table 2 summarizes previously reported cases of *K. rhizophila*-associated PD peritonitis [4-6]. Among adult cases, three have been reported, all requiring PD catheter removal due to relapsing peritonitis caused by *K. rhizophila* [4,5]. *Kocuria rhizophila* has been implicated in various infections; in catheter-related bloodstream infections, its ability to form biofilms has been hypothesized to promote recurrence [7-9]. Therefore, it is plausible that *K. rhizophila* could form biofilms on PD catheters, predisposing patients to relapsing PD-associated peritonitis. In this case, unlike previously reported adult cases, the peritonitis resolved without catheter removal. Notably, *K. rhizophila* was not detected in cultures of non-centrifugation PD effluent; it was identified only after centrifugation of PD effluent samples, as recommended by the ISPD guidelines. This suggests a lower bacterial load, allowing treatment to commence before forming the biofilm.

**Table 2:** Cases of PD-associated peritonitis caused by Kocuria rhizophila. ESRD: end-stage renal disease; PD: peritoneal dialysis.

Reference	Age/sex	ESRD cause	PD duration	Other bacteria cultured	Clinical findings	Antibiotic treatment	Catheter removal (reason)	Outcome
Nakata et al. [4]	78/Male	Diabetic nephropathy	Three years and eight months	None	Diarrhea, cloudy peritoneal fluid	Cefazolin ＋ ceftazidime ＋ vancomycin to penicillin G	Yes (relapsing peritonitis)	Cured
Lefur et al. [5]	70/Male	Nephroangiosclerosis	Five years	None	Abdominal pain, functional ileus, cloudy peritoneal fluid	Cefazolin ＋ ceftazidime to cefazolin to vancomycin	Yes (relapsing peritonitis)	Cured
Lefur et al. [5]	77/Female	Acute tubular necrosis	Seven years	None	Cloudy peritoneal fluid	Aztreonam ＋ vancomycin to vancomycin	Yes (relapsing peritonitis)	Cured
Ishihara et al. [6]	3/Female	Nephrotic syndrome	About two years	None	Abdominal pain, fever	Cefazolin ＋ ceftazidime	No	Cured
This case	63/Female	Chronic glomerulonephritis	One year	None	Abdominal pain, diarrhea, cloudy peritoneal fluid	Cefazolin ＋ ceftazidime	No	Cured

Regarding the route of infection, the patient had diarrhea before the onset of PD-associated peritonitis. Furthermore, CT scans showed signs of colitis, indicating bacterial translocation. The diarrhea symptoms improved promptly with treatment. However, there have been few reports of enteritis caused by *K. rhizophila*, and it is debatable whether this case of PD-associated peritonitis was caused by bacterial translocation. On the other hand, previous case reports have shown that *K. rhizophila* can colonize the skin, and the possibility that contact contamination caused PD-associated peritonitis in this case cannot be denied. The optimal choice of antibiotics for *K. rhizophila*-associated PD peritonitis has not been established. In this case, the initial therapy recommended by the ISPD guidelines, consisting of cefazolin and ceftazidime, proved effective and was continued for 21 days [1].

The identification of *K. rhizophila* is known to be challenging with conventional methods, suggesting that some cases of previously undiagnosed relapsing PD-associated peritonitis may have been attributable to this organism. However, the recent adoption of advanced technologies, such as MALDI-TOF mass spectrometry, has increased case reports of *K. rhizophila*-related infections [9]. Furthermore, it is anticipated that its recognition in PD-associated peritonitis will also continue to grow. Despite this progress, the optimal treatment strategy for *K. rhizophila*-associated PD peritonitis has not yet been established. Further accumulation and analysis of cases are needed to develop a standardized approach to managing this condition.

## Conclusions

In conclusion, we report a case of *K. rhizophila*-associated PD peritonitis that was resolved without PD catheter removal. While the risk of biofilm formation and subsequent relapsing peritonitis due to *K. rhizophila* necessitates careful monitoring, even after initial treatment, this case suggests that early intervention may enable successful resolution without relapse. With advancements in identification techniques for culture testing like MALDI-TOF mass spectrometry, more cases of PD-associated peritonitis caused by *K. rhizophila* are expected to be reported, and continued case accumulation will be beneficial.
